# Constraints on soluble aerosol iron flux to the Southern Ocean at the Last Glacial Maximum

**DOI:** 10.1038/ncomms8850

**Published:** 2015-07-23

**Authors:** T.M. Conway, E.W. Wolff, R. Röthlisberger, R. Mulvaney, H.E. Elderfield

**Affiliations:** 1 Department of Earth Sciences, University of Cambridge, Cambridge CB2 3EQ, UK; 2 British Antarctic Survey, High Cross, Madingley Road, Cambridge CB3 0ET, UK

## Abstract

Relief of iron (Fe) limitation in the Southern Ocean during ice ages, with potentially increased carbon storage in the ocean, has been invoked as one driver of glacial–interglacial atmospheric CO_2_ cycles. Ice and marine sediment records demonstrate that atmospheric dust supply to the oceans increased by up to an order of magnitude during glacial intervals. However, poor constraints on soluble atmospheric Fe fluxes to the oceans limit assessment of the role of Fe in glacial–interglacial change. Here, using novel techniques, we present estimates of water- and seawater-soluble Fe solubility in Last Glacial Maximum (LGM) atmospheric dust from the European Project for Ice Coring in Antarctica (EPICA) Dome C and Berkner Island ice cores. Fe solubility was very variable (1–42%) during the interval, and frequently higher than typically assumed by models. Soluble aerosol Fe fluxes to Dome C at the LGM (0.01–0.84 mg m^−2^ per year) suggest that soluble Fe deposition to the Southern Ocean would have been ≥10 × modern deposition, rivalling upwelling supply.

## Abstract (web summary)

Elevated deposition of bioavailable atmospheric iron may have enhanced carbon storage in the glacial Southern Ocean. Conway *et al*. apply a novel rapid-filtration technique to iron trapped in Antarctic ice cores and show that glacial soluble iron deposition was an order of magnitude greater than the modern.

## Graphical abstract

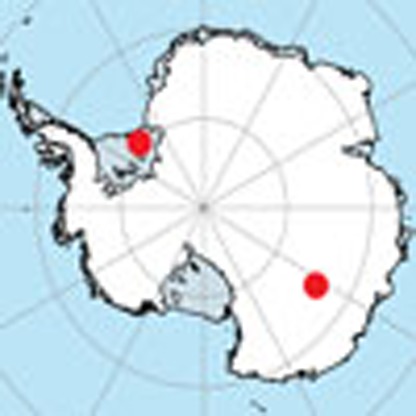

Changes in atmospheric iron (Fe) supply to the oceans (described here as aerosol Fe) during glacial intervals have been suggested as one of a number of possible drivers for changes observed in atmospheric CO_2_ between glacial and interglacial climate states (The Iron Hypothesis)^1^,^2^,^3^. Fe fertilization of the presently Fe-limited Southern Ocean during glacial times could be responsible for up to 40 p.p.m.v.^3^,^4^,^5^ of the regular 80–120 p.p.m.v. cycles in atmospheric CO_2_ that coincide with glacial–interglacial cycles over at least the last 800,000 years^6^. Increased atmospheric Fe supply to the glacial Southern Ocean, where today low dissolved Fe concentrations limit phytoplankton growth^4^,^7^, may have enhanced the efficiency of the ‘biological pump', retaining more carbon in the deep ocean and driving a reduction in pCO_2_ (refs 1, 2). While changes to ocean circulation and deep ocean carbon storage may play a dominant role in glacial–interglacial cycling^3^,^8^,^9^,^10^, atmospheric Fe may also strongly influence the efficiency of the biological pump on both glacial and millennial timescales^3^,^11^,^12^, with potentially large effects on the carbon cycle. Indeed, a recent study showed that increased nutrient utilization and greater productivity in the Sub-Antarctic are linked to periods of increased dust (and Fe) supply over the past 160,000 years^13^, highlighting atmospheric Fe fertilization in the Sub-Antarctic surface ocean as a candidate for explaining millennial-scale fluctuations in atmospheric CO_2_. Through this mechanism, Fe fertilization may play an important role in glacial–interglacial cycling, in combination with other processes^9^,^13^,^14^. Evidence for higher dust fluxes comes from Antarctic ice cores, which record marked increases in atmospheric dust deposition to Antarctica during colder intervals over the past 800,000 years^15^, most likely due to expanded dust source areas in South America and enhanced atmospheric lifetime and transport^16^. Sedimentary records also show that the temporal pattern of Antarctic ice-core dust fluxes is representative of dust reaching the Southern Ocean, with the dust–climate coupling extending back at least 4 million years^5^. However, modelling efforts to understand the sensitivity of the oceans and climate to Fe (for example, ref. 17) are still limited by poor constraints on aerosol Fe solubility and the fluxes of ‘bioavailable' (the Fe that can be taken up for growth by phytoplankton) aerosol Fe to oceans during glacial times.

Converting ice and sedimentary dust records to bioavailable aerosol Fe fluxes is not straightforward. Previous studies have used dust flux records with fixed composition and Fe solubility^18^, or non-seasalt Ca (nss-Ca) as proxies for dissolved Fe (ref. 11). Recently, measurements of Fe concentration and fluxes in ice cores have become available^19^,^20^,^21^,^22^,^23^; however, these were made under acidic conditions (pH 1–2), and are thus far-removed from dissolution in seawater (pH ∼8) where Fe solubility can be <1% (ref. 18). Fe solubility is strongly pH dependent (Supplementary Discussion), and acid leaches may access a variable fraction of the total Fe present^21^,^24^. Equally, dissolved Fe concentrations within melted Antarctic ice of glacial age (for example, 1–70 ng g^−1^ in this study) are typically well above the low-equilibrium saturation concentration of Fe(III) (<0.1 ng g^−1^; Supplementary Fig. 1), meaning that Fe loss due to precipitation/adsorption of Fe hydroxides is likely under weakly acidic pH conditions (Supplementary Discussion). While Fe(II) is more soluble than Fe(III) under these conditions^25^, a recent study suggests that a large proportion of Fe within ice-core material is present as Fe(III) (ref. 26). Any loss of Fe(III) via precipitation or adsorption to plastic equipment during processing that is not recovered during acidification will lead to underestimation of soluble Fe, which might affect studies analysing Fe after melting using discrete bottled samples or flow-through systems (for example, refs 19, 20, 23, 26). Thus, while previous ice-core records provide valuable information about broad-scale changes in aerosol Fe fluxes, they provide incomplete information about soluble or bioavailable aerosol Fe supplied to the oceans. We were therefore motivated to seek techniques for more representative estimates of soluble Fe flux to the Southern Ocean during glacial intervals to allow models to more accurately assess the effect of changing atmospheric Fe supply on the carbon cycle.

Here we present high-resolution fluxes of total and soluble atmospheric Fe, as well as calculated aerosol Fe solubility, from the Last Glacial Maximum (LGM; 21–26 kyr before present (BP)) of the EPICA Dome C ice core^15^,^20^ from East Antarctica (EDC; 75° 06′ S 123° 21′ E, 3,233 m.a.s.l.; Fig. 1) and from Marine Isotope Stage (MIS) 2-3 (22–52 kyr BP) from an ice core from Berkner Island (79° 32.9′ S 45° 32.9′ E; Fig. 1). Soluble Fe was determined using a novel rapid-filtration technique, in meltwater at pH ∼5.3 (the natural pH of the melted ice) or following leaching of sublimated dry dust with natural seawater, analogous to instantaneous techniques used with modern aerosols^27^. Our data are much more representative of dissolution of atmospheric dust in the ocean than previous ice-core studies, avoiding both unrepresentative leaching by strong acids^19^,^20^,^22^,^23^ or precipitation of insoluble Fe(III) hydroxides during melting. These methods are relevant for the possible modes of deposition of dust into the ocean, either deposition within rain, where dust has already been exposed to liquid at pH ∼5.3, or deposition of dry dust or solid ice/snow containing dust to the ocean where the first liquid encountered is seawater. We find that both total aerosol Fe fluxes and aerosol Fe solubility were very variable throughout the LGM in EDC ice (1–42% soluble; pH ∼5.3 mean 10%, median 6%) leading to highly variable soluble aerosol Fe fluxes of 0.01–0.84 mg m^−2^ per year at Dome C throughout the interval. At Berkner Island, although aerosol Fe solubility was lower than Dome C (mean ∼3%), higher dust fluxes resulted in similar mean soluble aerosol Fe fluxes to those at Dome C during the LGM. While these calculated soluble Fe fluxes are not fully representative of bioavailable Fe fluxes to the Southern Ocean, they enable an improved estimate of the atmospheric Fe that would have been deposited and dissolved in seawater at the LGM compared with previous studies. Our measured aerosol Fe solubilities and soluble aerosol Fe fluxes are higher than typically used in models of ocean biogeochemistry, and thus we suggest that models should consider higher and more spatially and temporally variable soluble atmospheric Fe fluxes to the Southern Ocean during glacial intervals.

## Results

### Figure summary

Aerosol Fe solubility and calculated total and soluble Fe fluxes from EDC and Berkner ice cores are shown compared with other ice-core parameters in Figs 2, 3, 4, with a comparison of Fe solubility in EDC ice of LGM age at pH ∼5.3 and in seawater shown in Fig. 2b. The data are also available in Supplementary Data.

### Aerosol Fe solubility in EDC and Berkner ice cores

**Figure 1:**
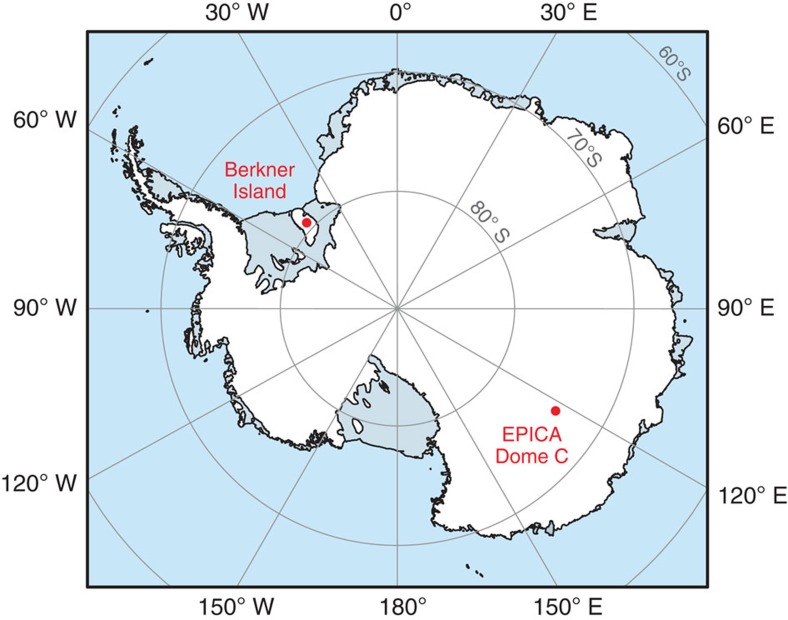
The locations of EPICA Dome C and Berkner Island coring locations on Antarctica.

**Figure 2:**
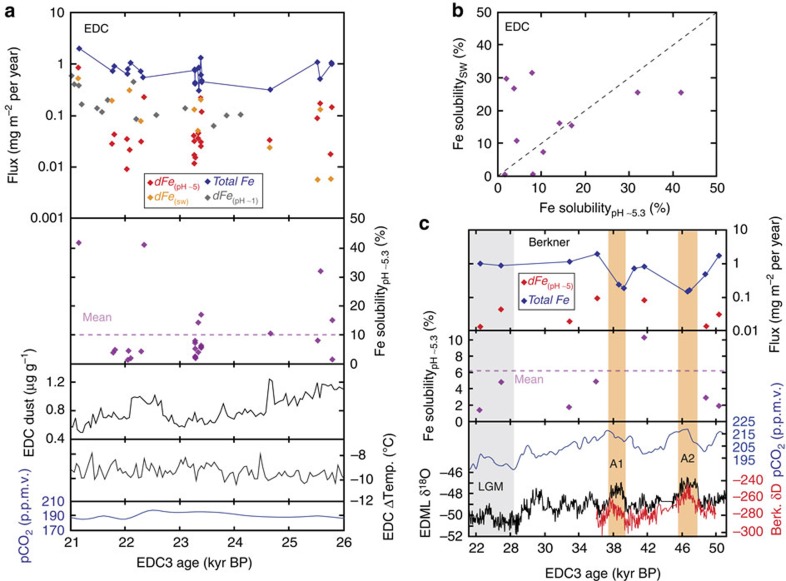
Variability in fluxes and chemistry of aerosol Fe deposited to Antarctica shown with other climatic parameters. (**a**) Fluxes of total and soluble (pH ∼5.3, seawater, pH ∼1 (ref. 20)) aerosol Fe, and Fe solubility across the LGM interval from the EPICA Dome C core. (**b**) Comparison of aerosol Fe solubility at pH ∼5.3 and in natural seawater (pH ∼8) from EDC ice of identical LGM age. Dashed line indicates 1:1. (**c**) Fluxes of total and soluble (pH ∼5.3) aerosol Fe and Fe solubility from MIS 2–3 of the Berkner Island Core. The grey bar denotes the LGM and the orange bars denote Antarctic warm events. Data points represent two measurements of a single sample, and errors are smaller than the size of points. All data are shown on the EDC3 age scale^47^. See Methods section for details of other climatic parameters, and details of conversion of Berkner age to EDC3.

Fe solubility in atmospheric dust deposited at Dome C across the LGM had a mean of 10% (*n*=23) and 17% (*n*=11) at pH ∼5.3 and in seawater, respectively, and a median of 6% at pH ∼5.3 (Fig. 2 and Supplementary Data). These aerosol Fe solubilities are higher than solubility of loess and desert dust (<0.5%; refs 18, 28) or glacial flour (2–3%; ref. 28), but consistent with high-end solubility estimates for modern atmospheric dust, where transport-induced size/compositional fractionation and chemistry enhance solubility away from source regions^29^,^30^. Indeed, dust dry-deposited^20^ at Dome C during the LGM represents the distal fine-fraction of dust leaving the source regions on Patagonia^31^. EDC dust is enriched in both Al and Fe compared with both the crust and Patagonian topsoil (Supplementary Data), suggesting that high Fe solubility at Dome C could be driven by a compositional bias towards smaller clay particles containing more readily soluble Fe^32^ during atmospheric transport^30^,^33^. By contrast, mean Fe solubility at Berkner Island, closer to South American dust sources^18^, was only 3% during 23–50 kyr BP (Fig. 2c) and 1–5% at the LGM.

**Figure 3:**
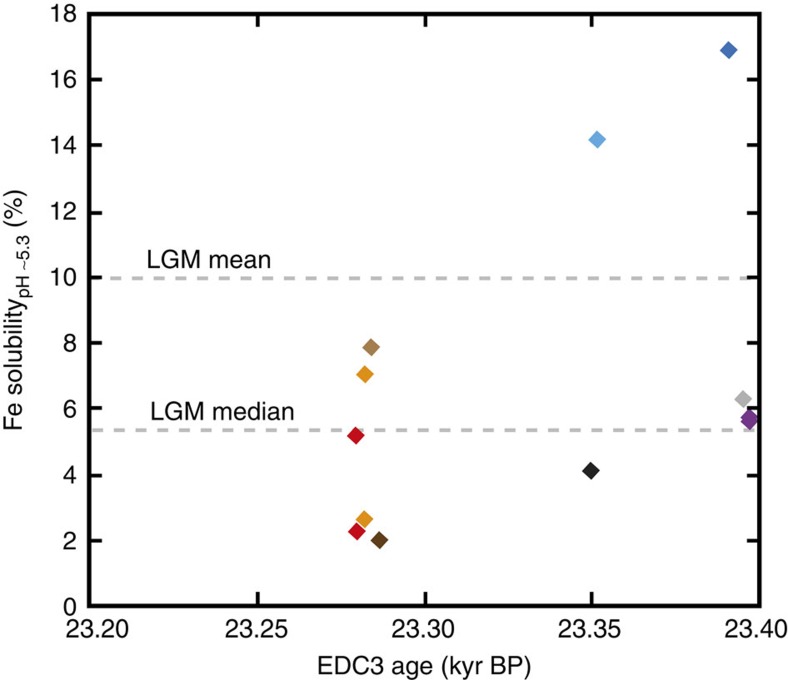
Fe solubility at pH ∼5.3 in EPICA Dome C ice of LGM age at high resolution. Ice of identical/similar age was subsampled (Supplementary Data) to investigate variability in Fe solubility. Ice samples of different age are shown in different colours, with three ice samples sampled twice (red 23.280 kyr BP, orange 23.282 kyr BP and purple 23.398 kyr BP; Supplementary Data). Data points represent two measurements of a single sample, and errors are smaller than the size of points. Dashed lines denote the mean and median of all EDC LGM measurements from Fig. 2.

**Figure 4:**
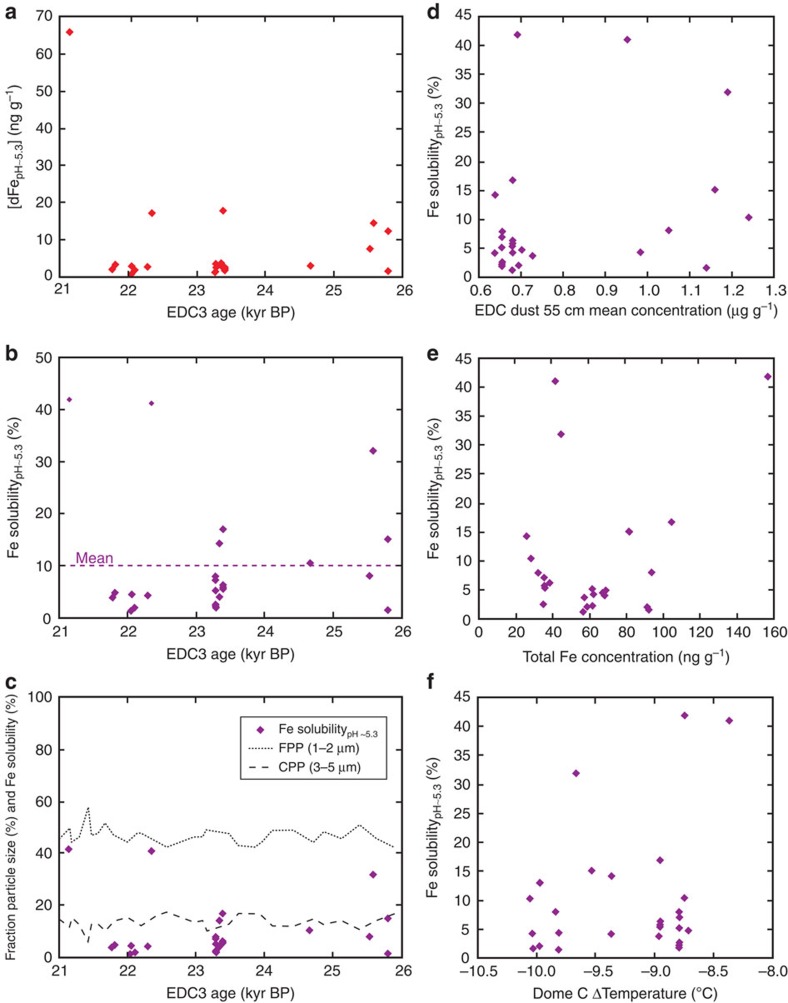
Variability in EPICA Dome C over the LGM. (**a**) pH ∼5.3 dissolved Fe concentration, (**b**) pH ∼5.3 Fe solubility and (**c**) pH ∼5.3 Fe solubility shown with grain size (fine particle, FPP; coarse particle, CPP) from coulter–counter analysis^15^,^70^. The relationship between pH ∼5.3 Fe solubility and (**d**) dust concentration^15^, (**e**) Total Fe concentration (this study), (**f**) Dome CΔ air temperature^68^. Data points represent two measurements of a single sample, and errors are smaller than the size of points.

Fe solubility in EDC samples was very variable across the interval (Fig. 2a; 1–42%). At several points within the LGM, we measured samples of very similar ages (Fig. 3), showing that variability sometimes also occurs at annual–decadal scales (Figs 2a and 3); for example, Fe solubility in samples at 23,350 and 23,352 kyr BP were 4 and 14%, and samples at 23,391 and 23,396 kyr BP were 17 and 6%, respectively (Supplementary Data and Fig. 3). Variability in Fe solubility through the LGM could be driven^24^,^29^ by seasonal changes in dust supply or discrete dust events, but is notable in ice samples, which by their nature represent combined dust deposition over several years. The observed variability in Fe solubility has no clear relationship with total Fe, dust loading or particle size (Fig. 4), suggesting that, if real, it is driven either by particle composition or transport-induced changes in Fe speciation. Similar EDC temperature (Fig. 2a) and assumed atmospheric transport pathways^16^, combined with a homogenous grain-size distribution, throughout the interval (Fig. 4), suggest that variability is more likely the result of compositional and Fe-speciation changes than transport-induced changes in Fe chemistry. Mineralogy exerts a strong control on Fe solubility, with Fe in clay and silicate minerals much more soluble than Fe hydroxides^32^, and glacial flour enriched in both Fe(II) and clays compared with loess^28^. While higher solubility may be driven by relative enrichment of clay minerals with transport^29^,^33^, variable solubility could be the result of changing contributions from different types of South American dust (for example, loess or glacial material^34^). Contributions from local dust sources is unlikely at Dome C, where most glacial-age dust is considered to be from Patagonia^31^, but they could play a role at Berkner Island.

Comparison of solubility at pH ∼5.3 and in seawater (Fig. 2b) also shows scatter about a 1:1 line, similar to that observed in modern studies comparing ultrapure water and seawater rapid-leaching techniques^27^. This scatter may reflect heterogeneous particle distribution and chemistry or could partly be the result of differences between the two methods, such as temperature, number of leaches, or solution chemistry enhancing dissolution in seawater. Similarly, while comparison of pH ∼5.3 leaching between three samples of identical age showed broadly similar solubility, there was also some variability (6 and 6%, 2 and 5% and 3 and 7%; Fig. 3). It is therefore possible that the full observed variability between samples in this study is not real; however, if anything, we would expect methodological concerns to result in underestimation of Fe solubility due to incomplete leaching or precipitation, which does not change our primary observation of high aerosol Fe solubility at times within LGM ice.

### LGM aerosol Fe fluxes at Dome C and Berkner Island

Decadal to centennial variability in both Fe solubility and total Fe fluxes throughout the LGM at Dome C (Fig. 2a; 0.3–2 mg m^−2^ per year; mean 0.76±0.4 mg m^−2^ per year) combines to mean that soluble Fe fluxes show great variability (Fig. 2a; 0.01–0.84 mg m^3^ per year), with mean values of 0.09±0.17 mg m^−2^ per year (1 s.d.) for pH ∼5.3 soluble Fe and 0.15±0.13 mg m^−2^ per year (1 s.d.) for seawater-soluble Fe. Despite the lower Fe solubility at Berkner Island compared with Dome C, higher atmospheric Fe fluxes at the LGM (0.95±0.07 mg m^−2^ per year), probably attributable to greater dust deposition owing to closer proximity to South American dust sources^18^, result in similar mean soluble Fe fluxes (0.03±0.02 mg m^−2^ per year) compared with those at Dome C. Total Fe fluxes at Berkner Island were almost an order of magnitude lower during Antarctic Warm Events A1 and A2 than during colder periods (Fig. 2c), reflecting lower regional dust fluxes during warmer intervals^15^.

## Discussion

The aerosol Fe flux data presented here can be compared with previous ice-core estimates of Fe fluxes to Antarctica and the nearby Southern Ocean, during the LGM. We find that our total EDC aerosol Fe fluxes (5.5–36 μmol m^−2^ per year; mean 13.7 μmol m^−2^ per year) are considerably higher than previous estimates based on acidic (pH ∼1–2) leaching or mineral dust proxies at several Antarctic locations (4.3–8 μmol m^−2^ per year; refs 22, 23), including those based on acidic leaches at EDC with a conversion to total Fe (refs 19, 22; 6.6 μmol m^−2^ per year). The difference may partially reflect the high-resolution variability in Fe flux at EDC, but also the fact that, similar to modern dust^24^, an acidic leach represents a variable portion of the total Fe, dependent on dust composition and leaching timescale. While pH ∼1 Fe EDC fluxes are generally higher than estimates at pH 5–8 (Fig. 2a), the two ranges overlap, meaning that acidic leaches of ice core do not represent either total or soluble aerosol Fe, although they may capture broad-scale trends. This offset between acidic leaches and total digested Fe may be partially to do with methodology; the pH 1 EDC Fe records were measured in samples that were melted, acidified and only left to dissolve for >24 h (refs 19, 20), while other studies have shown that Fe continues to dissolve at pH 1–2 for up to 3 months^22^,^35^. As a result, these short timescale acidic leaches in EDC ice samples may not have fully captured the pH 1 leachable fraction that is present. Previous comparison of pH 1 leached methods with digestion procedures on EDC ice suggested that 30–65% of total Fe was recovered by pH 1 leaching methods^19^,^21^, but it seems likely that the proportion accessed by these short-term acidic leaches would vary with dust composition and mineralogy. Also, even with 1–3 month leaching times, acidic leaches are unlikely to fully dissolve dust particles.

**Figure 5:**
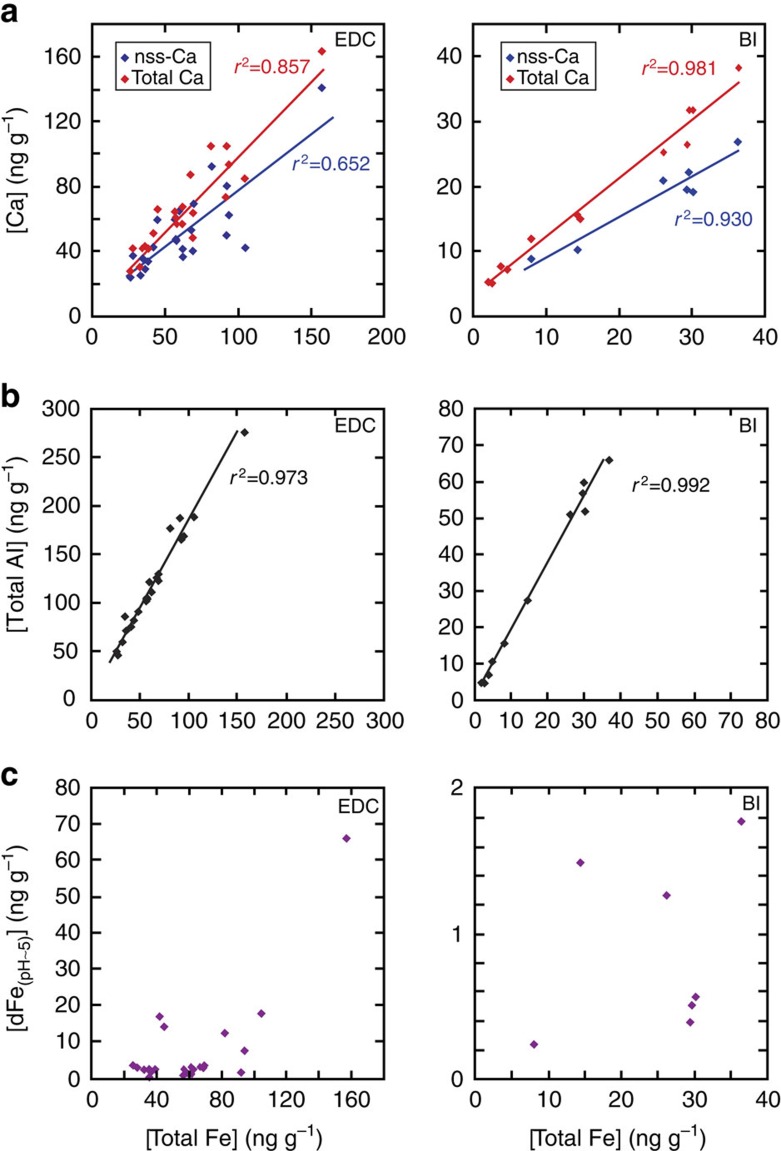
Direct comparison of different parameters as proxies for aerosol Fe in ice cores. Total Fe concentration, in EDC ice of LGM age (left panel) and Berkner Island (BI) ice from MIS 2–3 (right panel), is shown plotted against (**a**) nss-Ca and total Ca concentration, (**b**) total Al concentration and (**c**) pH ∼5.3 dissolved Fe concentration. Best-fit linear regression lines with *r*^2^ values are shown. Data points represent two measurements of a single sample, and errors are smaller than the size of points.

Our data also allow evaluation of the use of other proxies for ice core Fe (Fig. 5). We find that both nss-Ca^11^ (Ca with Na-calculated seasalt component removed) and total Al are good proxies for the flux of total Fe (Fig. 5). However, taking the EDC dust flux and calculating total Fe concentrations, assuming a constant crustal Fe percentage of ∼4% in dust^18^ underestimates total Fe for EDC samples, which are enriched in Fe (mean 8 wt%; Supplementary Data) compared with crustal values of 2–3% (ref. 36) or Patagonian topsoil (2–6%; ref. 37). EDC samples are similarly enriched for Al (mean 15 wt%; Supplementary Data), compared with crustal values of 8% (ref. 36) and Patagonian topsoil of 6–10% (ref. 37). This difference in the dust composition presumably relates to compositional-fractionation processes during transport, such as relative enrichment in silicate minerals, such as clays and feldspars^33^. As such, changes in composition and mineralogy may be spatially variable and site specific for ice-coring locations, complicating efforts to use dust, Ca or Al fluxes as proxies for total Fe. In addition, despite the possible usefulness of these proxies for providing information about timing and magnitude of changes in atmospheric Fe^11^, the lack of relationship between total and soluble Fe (Fig. 5c) means that they do not approximate soluble or bioavailable Fe. Instead, direct measurements of soluble Fe, together with biological experiments, are needed for constraining changes in soluble and bioavailable Fe supply to the oceans during climatic change and especially within stable climate intervals. However, we do note the strong covariance between ice-core dust and Fe fluxes and both Sub-Antarctic productivity and heavier Sub-Antarctic surface nitrogen isotope ratios (indicative of increased nutrient utilization) during the last 160 kyr (ref. 13), as well as a similar covariance between ice-core dust and (pH 1) Fe fluxes, sediment Fe mass accumulation rates and Sub-Antarctic export productivity over the last eight glacial cycles^5^,^38^; these relationships suggest that both dust or (pH 1 leached) EDC ice-core Fe fluxes do capture the order of magnitude changes in Fe supply to the Southern Ocean that are important for understanding millennial-scale climate and pCO_2_ variability.

By comparison with estimates of Holocene Fe fluxes to Antarctica^22^, our mean total Fe flux estimates (13.7 μmol m^−2^ per year) represent a ∼30–50 × increase over EDC (0.3–0.4 μmol m^−2^ per year), and a ∼10–20 × increase over coastal sites such as Law Dome (ref. 22; 0.8–1.1 μmol m^−2^ per year). The modern gradient in dust deposition between coastal sites and EDC is thought to be largely influenced by wet deposition, which dominates at coastal sites and is a minor factor inland, and thus might be somewhat smoothed out during drier glacial intervals^22^. In terms of soluble Fe, the mean EDC seawater-soluble Fe flux of 2.7 μmol m^−2^ per year represents a >10-fold increase over modern soluble Fe fluxes (even assuming a similar high solubility in modern dust), and is ∼10 × higher than modern calculated Southern Ocean atmospheric Fe fluxes (0.2 μmol m^−2^ per year; ref. 4). This suggests that the soluble atmospheric Fe flux to the Southern Ocean increased by at least a factor of 10 at the LGM. Such an Fe flux would be broadly equivalent to the expected upwelling supply of deep Fe, which would be reduced from calculated modern values of 8–16 μmol m^−2^ per year due to decreased circulation and stratification due to increased sea ice^2^,^4^. The range in soluble Fe fluxes observed in this study (0.2–15 μmol m^−2^ per year) is also similar to modelled modern upwelling Fe flux values for entrainment (deep winter mixing; 9.1–33 μmol m^−2^ per year) and vertical diffusion (1.2–3.6 μmol m^−2^ per year), which have been suggested to sustain surface water Fe concentrations in the modern Southern Ocean^39^.

Of course, upwelling of deep water supplies both Fe and carbon, and it is the proportion of this carbon that is utilized by primary productivity in surface waters, and thus retained in the ocean, which determines the surface Southern Ocean's role in setting pCO_2_. While the surface ocean is Fe-limited (insufficient bioavailable Fe to fully utilize all the upwelled nutrients), surface waters can act as a source for pCO_2_. Deposition of atmospheric dust only supplies Fe and thus provides the means to increase utilization of upwelled carbon and nutrients in surface waters. A full understanding of the role of atmospheric Fe supply in carbon cycling of the past thus requires not only knowledge of upwelling and dust deposition Fe fluxes but also the resulting change in the carbon to bioavailable Fe concentration ratio in surface waters, as well as how both Fe and carbon are recycled and/or taken to depth. However, models do suggest that an increase in bioavailable atmospheric Fe supply of 5–10 × could double export production^40^, meaning that the LGM EDC soluble Fe flux could be large enough to drive big changes in primary productivity, nutrient utilization and carbon sequestration, especially in the Sub-Antarctic zone of the Southern Ocean^9^,^13^. Although a recent ocean Fe biogeochemical modelling study^17^ suggested that pCO_2_ only has 2 p.p.m.v. sensitivity to dust globally, lower than the up to 40 p.p.m.v. suggested by recent Sub-Antarctic productivity/nutrient utilization records^13^, the model used a Fe solubility of only 2% (ref. 17), highlighting the need for constraints on soluble and bioavailable fluxes during glacial intervals.

In conclusion, our EDC soluble Fe fluxes suggest that soluble Fe fluxes to the Southern Ocean during the LGM and glacial times were likely higher than previously considered. Both Fe content (8 wt%) and mean Fe solubility (pH ∼5.3 10%; seawater 17%) in EDC dust are greater than typically assumed by models (∼1–10%; refs 17, 18, 41, 42, 43, 44), suggesting that models may underestimate soluble (and therefore bioavailable) Fe fluxes to the Southern Ocean at the LGM. This might especially be the case in modelling studies where 0.5–2% Fe solubility is used^17^,^41^,^42^,^43^. To address this, the median LGM pH ∼5.3 Fe solubility from this study (6%) is simplest for consideration in models, but the rapid variability in Fe fluxes on short timescales and higher solubilities should also be considered. We attribute variability in aerosol Fe solubility to changes in the composition and fluxes of atmospheric dust arriving at EDC, even on decadal timescales. Therefore, to fully capture the influence of atmospheric Fe on glacial–interglacial cycling, models must take into account both temporal and spatial variability in dust composition and Fe solubility. Closer to source regions, higher dust fluxes may offset lower Fe solubility, and short-term changes in the contribution of different dust types could have important effects on surface ocean biogeochemistry. We anticipate that these better general constraints on the soluble Fe flux to the Southern Ocean will be useful for modellers determining Fe's role in marine biogeochemical cycles over glacial–interglacial changes, as well as providing insight into the response of the oceans to anthropogenic CO_2_ release.

## Methods

### General laboratory procedures and equipment cleaning

All ice-preparation was carried out in a cold room (−20 °C) at the British Antarctic Survey, Cambridge, and all sample processing and analysis were carried out in clean laboratories at the University of Cambridge. Seawater analysis was carried out at Old Dominion University, USA. All clean work was carried out within a Class 1,000 HEPA-filtered clean laboratory and/or under Class 100 laminar flow air.

All water used was ultrapure (18.2 MOhm cm) and acids were either quartz-distilled (QD) to high purity from reagent grade or purchased as ultrapure (Fisher Scientific). Strict trace metal protocols were used, including wearing polyethylene (PE) gloves to minimize metal contamination during cleaning, processing and sampling. All plastic equipment was rigorously acid-cleaned following the procedures outlined below, which were based on previously published work^45^ or adapted from routine cleaning procedures in the Department of Earth Sciences at Cambridge.

New perfluroalkoxy (PFA) or fluorinated ethylene propylene (FEP) Teflon filter rigs, vials, jars, bottles and micro centrifuge tubes were cleaned by sequential immersion in weak (∼5%) Decon detergent (24 h), warm 50% (v/v) reagent-grade HNO_3_ (24–48 h) and warm 50% (v/v) reagent-grade HCl (24–48 h). Teflon equipment was re-cleaned by immersion in either warm 50% (v/v) HNO_3_ or HCl (24 h). All Teflon equipment was thoroughly rinsed with ultrapure water following each step and then left to dry on a clean drying rack under laminar flow. For any Teflon equipment that was to be in contact with ice or samples at near-neutral pH, the plastic was filled with ultrapure water to condition the surface. Nalgene low-density PE and polycarbonate (PC) bottles were cleaned following previously published cleaning methods for preparation of equipment for seawater sampling^45^: bottles were sequentially immersed in weak (∼5%) Decon detergent (48 h), 1 M reagent-grade HCl (48 h), 6 M reagent-grade HCl (48 h) and ∼1% (v/v) QD-HCl (>3 months), with extensive rinsing with ultrapure water between steps. Bottles were conditioned to neutral pH by filling with ultrapure water for 1 week before use. Whatman Nucleopore PC membranes for filtration (0.2 μm) were cleaned by immersion in 6 M QD-HCl (48 h) and then rinsed extensively with ultrapure water. Membranes were always handled with clean PFA tweezers to minimize contamination. Membranes were stored in and flushed with ultrapure water before use to condition the plastic to near-neutral pH for contact with ice samples. Pipette tips were either acid-cleaned^45^ with 50% (v/v) reagent-grade HCl (24 h) and rinsed with ultrapure water (seawater samples), or rinsed twice with 10% (v/v) QD-HNO_3_ and twice with ultrapure water before use (non-seawater samples).

For handling and decontamination of ice samples, Kyocera ceramic knives and polytetrafluroethylene (PTFE) Teflon boards were used. Knives were cleaned by sequential immersion in 6 M reagent-grade HCl (24 h) and 1 M reagent-grade HCl (24 h), followed by extensive rinsing with ultrapure water. PTFE boards were cleaned by immersion in 6 M reagent-grade HCl and then rinsed extensively with ultrapure water. Arm-length PE gloves for handling ice were cleaned by immersion in weak (∼0.01% v/v) QD-HCl (24 h) and rinsed thoroughly with ultrapure water to ensure no transfer of acid to samples.

### Ice samples and cutting and decontamination

Detailed information about the age of ice sampled in this study is shown in Supplementary Data. EDC samples from 21 to 26 kyr BP were chosen to cover the very high dust portion of the LGM (∼0.6–1.2 mg kg^−1^ dust; ref. 15) based on previous work^15^,^46^. ‘A cuts' (or ∼¼ of the core) from 15 separate ‘bags' of ice (55 cm vertical section; ∼50 years) from the EDC ice core were supplied by EPICA (see EPICA cutting plan in Supplementary Fig. 2a). For EDC sampling, ∼3 cm pieces were typically cut from either the bottom or top of each bag, using a steel bandsaw within the cold room (−20 °C) at the British Antarctic Survey. Two vertical cuts were then made to create three subsamples of identical age (Supplementary Fig. 2b), which were then taken for decontamination and processing to generate subsamples of identical age for total or soluble analysis. The sampling resolution was therefore typically 50 years, although each sample represents only ∼3 years. Four bags (959, 984, 986 and 987) were sampled at higher resolution (two to four 3 cm subsamples per 50 years) to investigate higher-resolution variability as well as reproducibility between ice subsamples of identical age. Precise age for each 3 cm sample was calculated from depth within each bag and the EDC3 age scale^47^. Berkner samples were chosen to cover MIS 2–3, with two samples within the LGM (23 and 26 kyr BP) and 10 samples across the ‘A' events^48^ of MIS 3 (36–50 kyr BP) based on the Berkner δD record (Mulvaney, unpublished data). Ice was supplied as ¼ pieces of Berkner core (diameter 50 mm), with 3 cm vertical pieces cut and subsampled analogous to EDC samples.

The outside of ice core material is typically unavoidably contaminated with trace metals from drilling, cutting with bandsaws and handling with gloves^49^. Thus, the outside of the ice must be decontaminated to remove outer layers where contamination overwhelms the natural signal. Previously, this has been carried out using a melt-head^50^, polyethylene lathe^51^, stainless steel blade^52^, ceramic blade or chisels^53^. Decontamination may require removal of several cm of core^22^,^51^,^52^,^54^ during low-dust or less-compacted intervals, or when elements of interest are only present at pg g^−1^ levels. Preliminary testing in this study demonstrated that, as expected, ultrapure water ice was severely contaminated following cutting with a bandsaw (20 ng g^−1^ Ca, 4 ng g^−1^ Fe and 8 ng g^−1^ Al). The small size of ice samples used in this study prevented the use of techniques that remove several cm of core, and so we developed a technique using Kyocera ceramic blades that sequentially removed contaminated ice-core material. Each piece of ice was placed on a PTFE board and held with a clean PE glove, while the outside few mm were scraped off with a clean ceramic blade. This was repeated three times using separate clean equipment. To achieve the lowest contamination levels, all ice sampling equipment and ice were only handled with clean PE gloves, and all ice decontamination was carried out under Class 100 laminar flow air within the cold room (−20 °C) at the British Antarctic Survey.

Decontamination blanks were 47 pg g^−1^ for Fe and 116 pg g^−1^ for Al (<0.1% of natural conc.), and testing established that using acid-cleaned equipment had no effect on the natural pH of ice (pH ∼5.3). Our simpler technique did not achieve the full decontamination of more rigorous chiselling methods^22^ (46 pg g^−1^ c.f. 4 pg g^−1^ Fe). However, our method can be applied to smaller pieces of ice, and the procedure reduces Fe contamination by factor of ∼100 × , resulting in insignificant contamination that represents <0.1% of typical natural Fe concentrations within ice from the high dust portion of the LGM.

### Total digestion and pH ∼5.3 rapid-filtration procedures

Following decontamination, ice samples for total digestion were allowed to melt within PFA vials and acidified with 50 μl of QD-HNO_3_ (conc.) per 5 ml. A 5-ml aliquot was evaporated to dryness, refluxed overnight at ∼80 °C with 100 μl QD-HNO_3_ (conc.) and 200 μl Teflon-distilled conc. HF, evaporated to dryness, redissolved in 100 μl of 6 mol l^−1^ QD-HCl, refluxed at ∼80 °C overnight and evaporated to dryness. Finally, samples were redissolved in 250–500 μl of 0.1 mol l^−1^ QD-HNO_3_ for analysis by inductively coupled plasma optical emission spectrometry (ICP-OES). Procedural blanks (*n*=26) were 1.9 ng g^−1^ (Fe), 5.7 ng g^−1^ (Al) and 2.8 ng g^−1^ (Ca), all <0.5% of natural concentrations.

Following decontamination, ice samples for soluble analysis were processed by either ‘rapid-filtration' (melting at natural pH, referred to as ‘pH ∼5.3) or ‘seawater-leaching' methods (see later sections). Ice samples for rapid filtration were allowed to melt at room temperature (∼2 h) on a 0.2-μm Whatman polycarbonate membrane inside a 47-mm PFA Savillex filter rig, under vacuum. Rigs were covered to prevent contamination. Liquid that formed was quickly filtered and collected in a PFA reservoir containing an appropriate volume of conc. HNO_3_ to acidify samples to pH 0. On completion of melting, dust was leached with three 25 ml aliquots of ultrapure water, similarly acidified. This procedure, analogous to instantaneous leaches of modern aerosols^27^, was designed to minimize any FeOH_3_ precipitation or wall adsorption, which are concerns with flow-through and long leaching procedures (Supplementary Discussion). Acidified samples were evaporated to dryness and redissolved in 300 μl of 0.1 mol l^−1^ QD-HNO_3_ for analysis by ICP-OES. Fe concentration from the meltwater and subsequent ultrapure leaches were combined in final calculations. Procedural blanks (*n*=78) were 17 pg g^−1^ (Fe), 244 pg g^−1^ (Ca) and 83 pg g^−1^ (Na), all corresponding to 0–2% of natural concentrations.

### Elemental analysis

Samples from total digestion or rapid filtration were analysed for dissolved Al, Ca, Fe and other cations using a Varian Vista ICP-OES at the University of Cambridge. Each sample was analysed six times and a mean concentration was determined by reference to a quadratic calibration curve based on five standards. Single-session accuracy on a 250-ng per gram internal multi-element reference standard for Al, Ca and Fe was >99.6% with precision of 0.41% (1 s.d.; *n*=10). Long-term reproducibility of the standard was 100±1% for all elements (*n*=60). Detection limits for Fe were 0.1–0.4 ng g^−1^. We assign 5% uncertainty on data to account for weighing, pipetting and %RSD of six ICP-OES analyses (typically <5%).

### Sublimation

Sublimation allows the extraction of gases or dust from ice-core material while preventing melting, which could compromise the sample fidelity, and is achieved by lowering the pressure and temperature of ice below the triple point (0.01 °C, 6.1173, mbar). Sublimation has been previously used to investigate concentrations and isotope ratios of gases (for example, CO_2_ and CH_4_) released from bubbles trapped in ice-core material^55^,^56^,^57^,^58^, as well as having been utilized to cross-check CO_2_ measurements obtained using other techniques in Bern^59^. In the present study, we were motivated to develop a sublimation technique to extract dust from ice cores without exposing the dust to liquid water at a pH lower than that of seawater (pH ∼8), which may alter the speciation or solubility of Fe within dust before analysis (Supplementary Discussion). The major mode of deposition of dust at Dome C during the LGM is thought to be dry^20^, meaning that EDC dust extracted by sublimation should be representative of dry dust deposition at Dome C during the LGM.

We designed a sublimation apparatus based on previous studies on gases^58^, adapted to cleanly extract large masses of dust^57^ from ice without melting. The apparatus consists of a Schott borosilicate glass desiccator, sealed with Apiezon N vaccum grease, a vaccum pump with oil cleaner and pressure gauge and a −100 °C freeze trap for removal of water vapour (Supplementary Fig. 3). To allow flexibility depending on the mass of dust/ice required by experiments, two configurations of the system were designed (Supplementary Fig. 3). In the present study, ice samples were placed directly on membranes within filter rigs within a Savillex PFA Teflon Jar with top ports open (25–50 g; Supplementary Fig. 3a). A second configuration, not used in the present study, allows for the collection of large dust masses, with larger pieces of ice (>50 g) placed within 1 l bottles within a 4-l bottle with holes drilled in the lid (Supplementary Fig. 3b). Although ice sublimes under any pressures and temperatures below the triple point, previous studies have shown that quasi- and surface-melting can occur on ice or ice–glass surfaces at temperatures (−1 to −4 °C) and pressures below the triple point^60^,^61^,^62^. To avoid this, it is necessary to keep the ice cold until the pressure is sufficiently lowered, and to operate at pressures much lower than the triple point (<2 mbar). To achieve this, first the glass desiccator was taken into a Class 100 laminar flow bench within the cold room and allowed to cool before being cleaned with propanol. Jars or bottles were then placed within the cleaned desiccator and the desiccator was closed and sealed before being transferred to the warm laboratory and connected to the pumps. This ensured that samples remained clean and that the ice remained cold for ∼10 min while the pressure was reduced below the triple point to the typical running pressure of 0.8–1.6 mbar.

Sublimation was allowed to proceed until all ice was gone, which was assessed visually or when the pressure had dropped to the background of the empty chamber (0.26 mbar). Rates of sublimation were relatively slow, dependent on ice mass and volume, with a maximum rate of ∼250 g per 24 h. Small samples (5–10 g) sublimed in several hours, but were typically left overnight to ensure all ice was gone.

Once all ice had sublimed, the desiccator was sealed, disconnected and returned to the laminar flow bench to slowly allow clean air to enter the chamber; this prevented both dust being blown away by a sudden increase in pressure and the entry of dirty air from the pump. The bottles containing the dry dust samples were then carefully bagged and taken for ‘seawater-leaching' procedures (see next section). Blank contamination for the whole procedure over 24 hours was found to be acceptably low (∼40 pg Fe per ice sample).

### Seawater-leaching procedures and Fe analysis

Ice samples for seawater-soluble Fe analysis (‘seawater-leaching') were placed on a membrane on a PFA rig within a 1 l PFA jar within a glass desiccator at −20 °C, and quickly placed cleanly under vacuum (before moisture could form) at ∼18 °C. Samples remained under vacuum until all the ice had sublimed, leaving dry dust on the membrane (see ‘Sublimation' section). The dust on the membrane was then leached with four 60-ml aliquots of low-Fe Ross Sea seawater at 2 °C. The Ross Sea seawater used in leaching was previously collected^63^ from 10 m depth in the Southern Ross Sea (76° 02′ S, 169° 53′ E) under clean conditions in December 2003. The water was filtered with 0.2 μm polypropylene cartridges and stored in 125 l polyethylene barrels, before being subsampled for this work using clean procedures^63^. We determined the background dissolved Fe concentration in this seawater to be 4.7 pg g^−1^ (0.11±0.02 nmol l^−1^, 2 s.d.; *n*=4), consistent with previous determination^63^,^64^ of 0.07±0.02 nmol l^−1^. Each aliquot of seawater was allowed to leach the dust momentarily, vacuum was applied and then the liquid was pulled through and acidified to pH 1.6 with 120 μl of conc. ultrapure HCl. Samples were left acidified for >2 weeks before being analysed for dissolved Fe by FIA-8HQ, following published techniques^45^. This method has low detection limits (<5 pg g^−1^) and analytical precision is typically 5–10% during routine analysis; we therefore applied 10% uncertainty on seawater Fe concentration. Fe concentrations from four leaches were combined in final calculations.

### Berkner Deuterium and parameters and age scales

The ice core at Berkner Island was drilled to bedrock, and completed in 2005 (ref. 65 and Fig. 2). Deuterium (δD) ratios (expressed relative to the international standard Vienna Standard Mean Ocean Water) were measured at the NERC Isotope Geosciences Laboratory using standard methods^66^ and have a typical precision of 1.0‰. In Fig. 2b, the Berkner δD record across A events A1 and A2 is shown overlain on the EPICA Dronning Maud Land (EDML) δ^18^O record^67^, which is shown on the EDML1 age model that has been synchronized to EDC3 (refs 47, 67). In the absence of an official age model for this part of the Berkner Island ice core, Berkner δD is approximately visually tuned to the EDML δ^18^O record so that EDML δ^18^O, Berkner δD and Berkner Fe data are all shown on approximately the EDC3 age scale. Dust and temperature in Fig. 2a are from the EDC core^15^,^68^, while atmospheric CO_2_ concentration (pCO_2_) in both Fig. 2a,b is taken from the composite CO_2_ record of Schilt *et al*.^69^

### Fe solubility and flux calculation

Elemental fluxes were calculated:


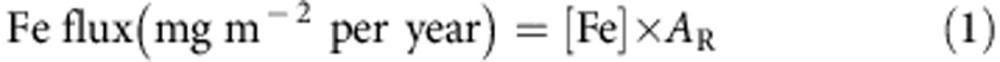


where [Fe] is the concentration of Fe (mg kg^−1^) in the ice, *A*_R_ is the calculated ice-accumulation rate (kg m^−2^ per year; ref. 47, Mulvaney, unpublished data).

Fe solubility was defined:


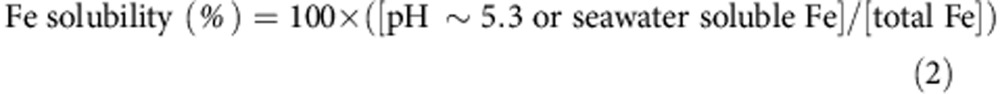


where [pH ∼5.3 or seawater-soluble Fe] and [total Fe] are the concentration in ice meltwater (ng g^−1^), combined from multiple leaches.

### Calculating nss-Ca

nss-Ca in each ice-core sample was calculated in this study from measured dissolved Ca and Na concentration data using paired simultaneous equations^50^:


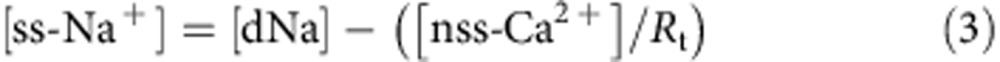



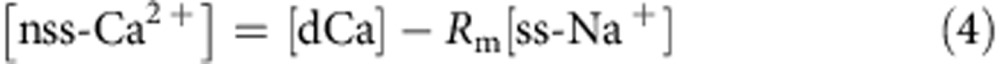


where ss denotes from seasalt, nss from non-seasalt sources, [dNa] dissolved Na concentration at pH ∼5.3, [dCa] dissolved Ca concentration at pH ∼5.3, *R*_t_ and *R*_m_ are the Ca/Na ratios in crustal material (1.78) and seasalt (0.038), respectively^50^.

## Additional information

**How to cite this article:** Conway, T. M. *et al*. Constraints on soluble aerosol iron flux to the Southern Ocean at the Last Glacial Maximum. *Nat. Commun.* 6:7850 doi: 10.1038/ncomms8850 (2015).

## Supplementary Material

Supplementary Information Supplementary Figure 1-3, Supplementary Discussion and Supplementary References

Supplementary Data 1 Ice core dataset
