# Supplementary material for: Constraints on soluble aerosol iron flux to the Southern Ocean at the Last Glacial Maximum
Source: Nat Commun. 2015 Jul 23;6:7850. doi: 10.1038/ncomms8850 (PMC4525188; doi:10.1038/ncomms8850)
Supplement: Supplementary Information — Supplementary Figure 1-3, Supplementary Discussion and Supplementary References [file ncomms8850-s1.pdf]

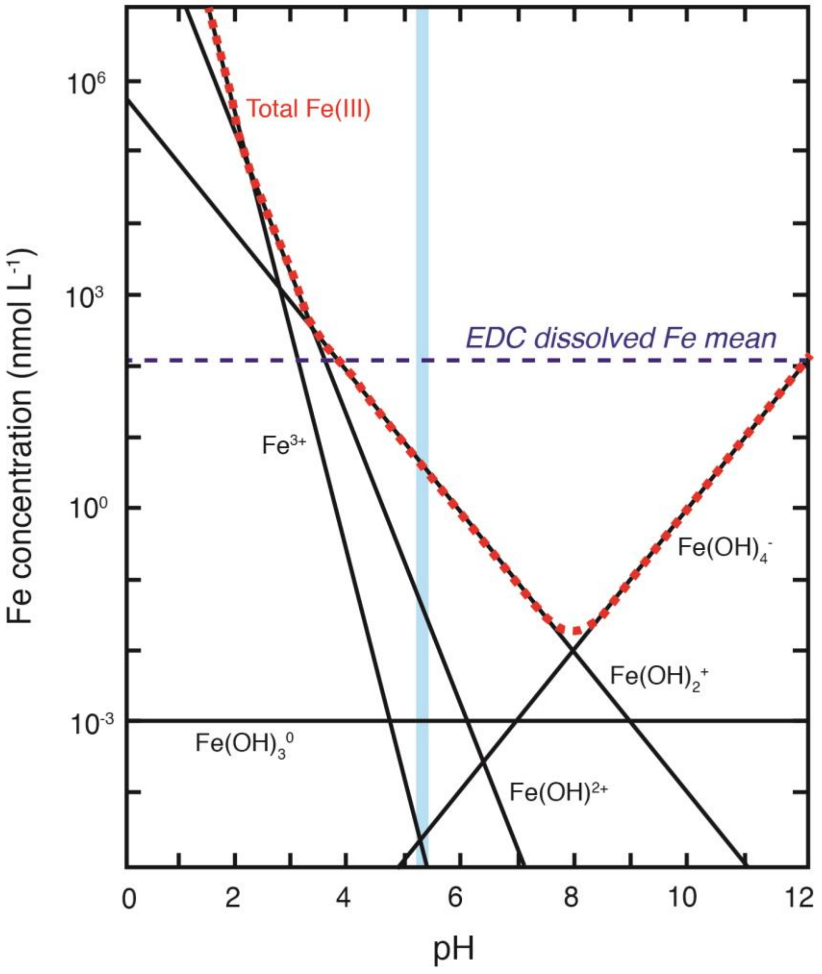

Supplementary Figure 1. Fe speciation and solubility in aqueous systems as a function of pH. Theoretically calculated solubility of different Fe species of Fe(III), (redrawn from Stumm, Morgan and Drever[4]). Dashed red line shows total theoretically calculated dissolved Fe(III) and dashed blue horizontal line denotes the mean dissolved Fe concentration from EDC ice at pH 5 from this study (7 ng g^{-1}). The light blue shaded vertical bar represents the pH of EDC ice samples measured in this study (5.2-5.4).

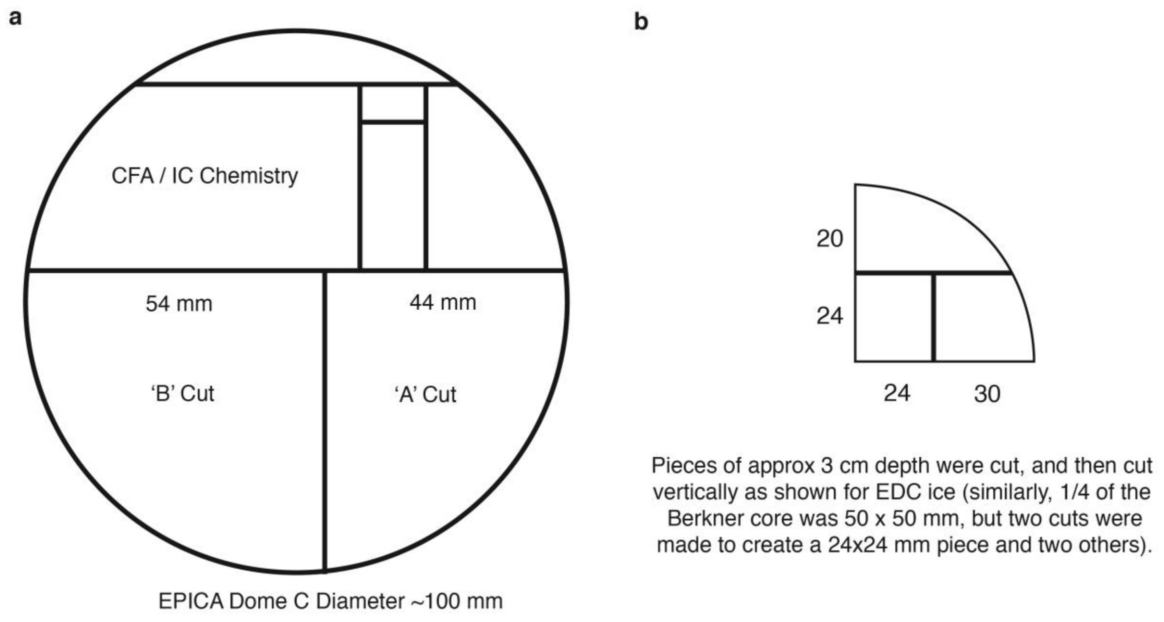

Supplementary Figure 2. Ice Cutting Plan. a. EPICA ice-core cutting plan, showing 'A' cuts used in this study. b. Ice subsampling plan used in this study for both EDC and Berkner samples.

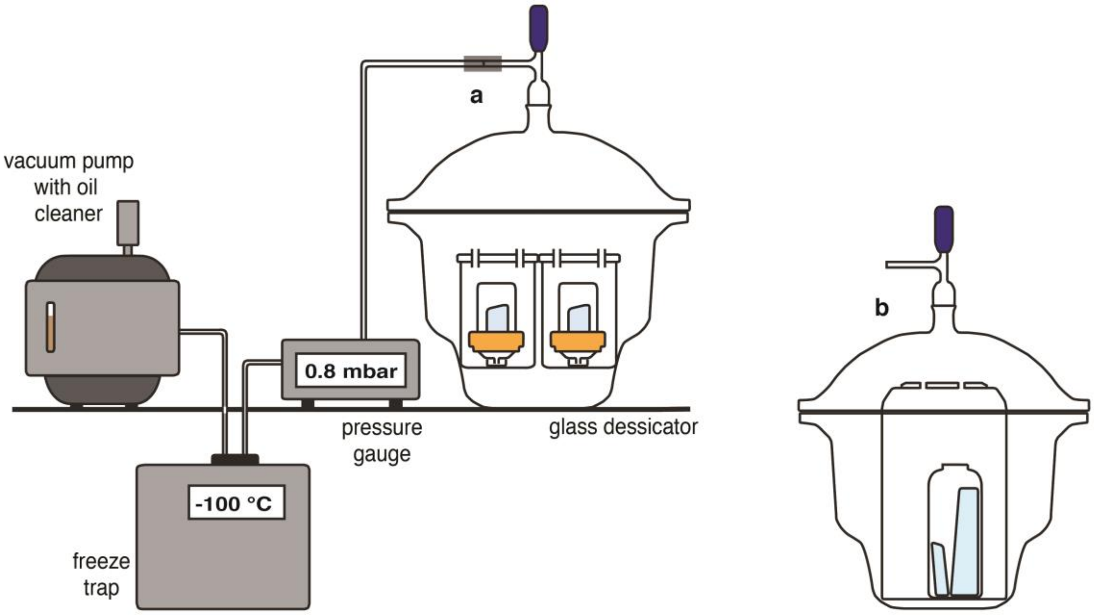

Supplementary Figure 3. Sublimation apparatus used in this study. Two possible configurations are shown: a. for subliming small ice samples on filtration rigs for solubility analysis (used in this study), **b.** possible configuration for subliming larger ice samples to collect large masses of dust.

Supplementary Discussion

Accurate analysis of ice-core and other aqueous material for dissolved elemental concentrations relies on an understanding of the chemical properties of these elements. For example, for metals such as calcium or sodium, which have high saturation concentrations in aqueous solutions, and are therefore not easily precipitated during processing, melting and analysis of ice cores using a melt-head and flow through system is suitable[1,2]. Such a system has the advantage of decontamination being included during processing[1,2]. However, when the focus of study is particle-reactive insoluble elements such as Fe from aerosol dust, the solubility, physiochemical speciation and pH in solution must be taken into consideration when devising methods.

The form and presence of dissolved iron (Fe) in aqueous or saline solutions depends strongly on pH, with dissolved Fe above pH 3-4 in oxic conditions being present principally as Fe(III) associated with hydroxide (OH^-) complexes[3,4]. As pH rises, Fe(III) complexes progressively[3] with OH^- to form $FeOH^{2-}_{(aq)}$, $Fe(OH)_2{}^-_{(aq)}$ and $Fe(OH)_{3(s)}$. These complexes may then be precipitated or adsorbed to plastic walls, compromising the accuracy of dissolved Fe measurements, as is well known from seawater studies[5,6]. In weakly acidic melted ice-core material, at pH ~5.3 (EDC ice measured between 5.2 and 5.4 in this study), Fe(III) has a low equilibrium saturation concentration[3,4] (<0.1 ng g^{-1}; Supp. Fig. 1), as aqueous conditions are close to those where formation of the solid ferrihydrate is favoured over dissolved Fe species[3]. Dissolved Fe concentrations in melted EDC LGM ice are generally much higher (0.7-66 ng g^{-1}, mean 7 ng g^{-1}; Supp. Fig. 1) than the low saturation concentration, and samples are therefore predisposed to Fe loss from solution via adsorption to plastic bottles and dust particles, as well as precipitation as ferric hydrides, during melting. While Fe(II) has a much higher saturation concentration under these conditions[7], over the timescale of melting, the Fe(II) present may be oxidised and precipitated from solution, with ~20% of Fe(II) expected to be oxidized at pH 5 within 2 hours[7].

It is necessary to consider these processes that may complicate efforts to make accurate estimates of soluble Fe in ice-core material. To investigate this we carried out some simple experiments[8] with an 10,000 µg g^{-1} Fe(III) nitrate solution, with analysis of Fe by ICP-OES as described in Methods. As might be expected, we found that reliable measurement by ICP-OES of added dissolved Fe(III) at pH >3 to be compromised. We diluted the Fe(III) nitrate solution to 5-50 ng g^{-1} with UPW (to pH 4-5.5) and found

that evaporation without prior-acidification, filtration of a dilution older than 10 minutes, or simply the solution being left for >10 minutes all led to a measurable reduction in measured dissolved Fe in solution[8]. This reduction in measurable Fe was likely due to the formation of $FeOH_x$ complexes that either precipitate or interact with bottle walls, effectively removing them from solution. Similar processes would be expected to affect dissolved Fe(III) in ice samples as they melt, and could affect dissolved Fe measurements made with continuous flow analysis or with large ice samples allowed to melt at ambient temperatures. Indeed, in our experiments over the typical timescale of ice melting (several hours) measureable Fe(III) in solutions of 5-20 ng g^{-1} dropped by as much as 75%, very similar to lower-concentration studies in seawater[6,8]. Some of the missing Fe in our experiments (up to 80%) was later recovered from bottle walls by leaching with strong acids[8], demonstrating that precipitation and/or adsorption was at least partly responsible.

Incomplete knowledge of initial dust composition and Fe speciation, together with exact redox conditions preclude accurate prediction of the behavior of dissolved Fe(III) or Fe(II) as ice melts. Experimental evidence and theoretical predictions suggest that exposure of aerosol dust to liquid, releasing relatively high concentrations of Fe for more than a few minutes, is likely to result in a loss of dissolved Fe(III) from solution to either particles or walls. Recent work[9] showed that a large fraction of pH 1 leachable Fe in ice-core material was present as Fe(III), with the proportion of Fe(III):Fe(II) dependent on particle size[9] and presumably dust composition. Any loss of dissolved Fe(III) prior to filtration and measurement will lead to an underestimation of true Fe solubility in the samples. Methods designed to accurately measure dissolved Fe in ice-core material must therefore take steps to reduce this Fe loss during processing. There are two approaches that can be used: 1) Measurement at low pH so that any dissolved Fe will be retained in solution, or 2) Rapid leaching of dust so that any melt formed or liquid used are filtered and acidified prior to precipitation of Fe hydroxides.

At lower pH, the higher saturation concentration for Fe(III) (Supp. Fig. 1) should stabilize Fe in solution and reduce or prevent Fe loss to plastic equipment or particles. Accordingly, in experiments, we found that acidifying Fe(III) solutions to below pH 3.5 did alleviate the issue; after 4 hours, 80-100% of the initial Fe remained in solution. In fact, measurement at low pH is a technique that has been deployed in ice-core studies previously, with a 'total dissolvable Fe' at pH ~1-2 parameter being used by a number of studies[9–13]. However, there are some drawbacks with this approach, as while this parameter is often

used to represent total or dissolved Fe, such a strong acid leach is likely to capture a compositionally-variable portion of total or soluble Fe[14,15], especially when only short leaching times are employed[10]. For example, previous Fe studies of EDC ice and Talos Dome ice (at pH ~1) used acidification times of less than one week[9,11–13,16], but it has been shown that Fe and other metals continue to dissolve from dust particles for up to 3 months in ice-core samples[10,17], and a one month leach of modern aerosols at pH 2 captured a highly variable portion of total Fe in different samples from Bermuda[14]. Mineralogy and dust composition may influence the proportion of total Fe that is leached by these weak acid leaches, as well as the time taken to dissolve all pH 1 dissolvable Fe. This complicates both understanding of the pH 1 acid leached Fe fraction in these studies, as well as comparing different acid leachable Fe records[17]. Additionally, aerosol Fe dissolution is enhanced with declining pH[18,19]. Thus, while these methods may prevent under-representation of Fe solubility due to Fe precipitation, these techniques may also over-represent soluble Fe due to enhanced dissolution of Fe-bearing minerals. Later acidification may also not address losses of Fe to plastic tubing and plastic containers during processing, prior to acidification. If lost Fe is not recovered during acidification, this may be another complication for studies using melted-ice from continuous flow systems or in discrete bottled studies (e.g. refs 9,11–13,16).

Instead, we chose to take the second approach to avoid soluble Fe loss during processing and developed a rapid-filtration procedure where never-previously-melted ice was allowed to melt and then the liquid filtered instantly via vacuum filtration, analogous to instantaneous leaching of modern aerosols[20]. This rapid-filtration technique should prevent or reduce formation of insoluble Fe hydroxides that would remove Fe from solution over the course of melting. Momentary leaching of dust with 2°C seawater should have a similar affect, as well as reducing oxidation of Fe(II) in solution[7], also preventing Fe loss prior to analysis. Once filtered, the sample was immediately acidified to pH ~0 with clean concentrated HNO_3 to prevent any loss of Fe via adsorption to plastic walls or during evaporation for pre-concentration of samples. The strength of this new technique is that it acts both to reduce under-representation of Fe solubility due to Fe loss during processing, and generates Fe solubility estimates at a pH that are much more representative of solubility in seawater.

Supplementary References

1. Röthlisberger, R. *et al.* Dust and sea salt variability in central East Antarctica (Dome C) over the last 45 kyrs and its implications for southern high-latitude climate. *Geophys. Res. Lett.* **29,** 1963 (2002).

2. Kaufmann, P. R. *et al.* An improved continuous flow analysis system for high-resolution field measurements on ice cores. *Environ. Sci. Technol.* **42,** 8044–50 (2008).

3. Drever, J. I. *The geochemistry of natural waters*. (Prentice Hall, 1997).

4. Stumm, W., Morgan, J. J. & Drever. *Aquatic Chemistry*. (Wiley, 1996).

5. Johnson, K. *et al.* Developing standards for dissolved iron in seawater. *Eos Trans. AGU.* **88,** 131–132 (2007).

6. Fischer, A. C., Kroon, J. J., Verburg, T. G., Teunissen, T. & Wolterbeek, H. T. On the relevance of iron adsorption to container materials in small-volume experiments on iron marine chemistry: ^{55}Fe-aided assessment of capacity, affinity and kinetics. *Mar. Chem.* **107,** 533–546 (2007).

7. Morgan, B. & Lahav, O. The effect of pH on the kinetics of spontaneous Fe(II) oxidation by O_2 in aqueous solution - basic principles and a simple heuristic description. *Chemosphere* **68,** 2080–4 (2007).

8. Conway, T. M. Solubility and bioavailability of iron from dust in Antarctic ice cores. *Ph.D. Thesis.* University of Cambridge (2010).

9. Spolaor, A. *et al.* Iron speciation in aerosol dust influences iron bioavailability over glacial-interglacial timescales. *Geophys. Res. Lett.* **40,** 1618–1623 (2013).

10. Edwards, R., Sedwick, P. N., Morgan, V. & Boutron, C. Iron in ice cores from Law Dome: A record of atmospheric iron deposition for maritime East Antarctica during the Holocene and Last Glacial Maximum. *Geochemistry, Geophys. Geosystems* **7,** Q12Q01 (2006).

11. Gaspari, V. *et al.* Atmospheric iron fluxes over the last deglaciation: Climatic implications. *Geophys. Res. Lett.* **33,** L03704 (2006).

12. Wolff, E. W. *et al.* Southern Ocean sea-ice extent, productivity and iron flux over the past eight glacial cycles. *Nature* **440,** 491–6 (2006).

13. Vallelonga, P. *et al.* Iron fluxes to Talos Dome, Antarctica, over the past 200 kyr. *Clim. Past* **9,** 597–604 (2013).

14. Tian, Z., Ollivier, P., Véron, A. & Church, T. M. Atmospheric Fe deposition modes at Bermuda and the adjacent Sargasso Sea. *Geochemistry, Geophys. Geosystems* **9,** Q08007 (2008).

15. Ruth, U. *et al.* Proxies and Measurement Techniques for Mineral Dust in Antarctic Ice Cores. *Environ. Sci. Technol.* **42,** 5675–5681 (2008).

16. Spolaor, A., Vallelonga, P., Gabrieli, J., Roman, M. & Barbante, C. Continuous flow analysis method for determination of soluble iron and aluminium in ice cores. *Anal. Bioanal. Chem.* **405,** 767–74 (2013).

17. Koffman, B. G., Handley, M. J., Osterberg, E. C., Wells, M. L. & Kreutz, K. J. Dependence of ice-core relative trace-element concentration on acidification. *J. Glaciol.* **60,** 103–112 (2014).

18. Spokes, L. J., Jickells, T. D. & Lim, B. Solubilisation of aerosol trace metals by cloud processing: A laboratory study. *Geochim. Cosmochim. Acta* **58,** 3281–3287 (1994).

19. Zhuang, G., Yi, Z., Duce, R. A. & Brown, P. R. Chemistry of iron in marine aerosols. *Global Biogeochem. Cycles* **6,** 161–173 (1992).

20. Buck, C. S., Landing, W. M., Resing, J. A. & Lebon, G. T. Aerosol iron and aluminum solubility in the northwest Pacific Ocean: Results from the 2002 IOC cruise. *Geochemistry Geophys. Geosystems* **7,** 21 (2006).
